# The Effect of sequential bilateral low‐frequency rTMS over dorsolateral prefrontal cortex on serum level of BDNF and GABA in patients with primary insomnia

**DOI:** 10.1002/brb3.1206

**Published:** 2019-01-04

**Authors:** Jie Feng, Qing Zhang, Chengliang Zhang, Zhongmin Wen, Xianju Zhou

**Affiliations:** ^1^ Department of Neurology The Second Affiliated Hospital of Soochow University Suzhou China; ^2^ Laboratory of Neurological, Department of Neurology, Changzhou No.2 People’s Hospital The Affiliated Hospital of Nanjing Medical University Changzhou China; ^3^ Department of Neurology, Integrated Hospital of Traditional Chinese Medicine Southern Medical University Guangzhou China

**Keywords:** brain‐derived neurotrophic factor, primary insomnia, repetitive transcranial magnetic stimulation, γ‐aminobutyric acid

## Abstract

**Objective:**

This study aimed to investigate the effect of sequential bilateral low‐frequency repetitive transcranial magnetic stimulation (rTMS) over dorsolateral prefrontal cortex (DLPFC) on patients with primary insomnia (PI).

**Methods:**

A total of 32 eligible right‐handed participants diagnosed by PI according to International classification of sleep disorders (ICD‐3) were recruited into this study. Participants received 10 daily sessions of sequential bilateral 1 Hz rTMS over DLPFC. Before and after the whole procedure of rTMS, patients were assessed by Pittsburgh Sleep Quality Index (PSQI) for the severity of sleep disturbance. Meanwhile, serum concentration of brain‐derived neurotrophic factor (BDNF) and gamma‐aminobutyric acid (GABA) in patients was measured by ELISA and UPLC, respectively. Moreover, the amplitude of MEPs reflecting the right cortical excitability was examined. Finally, Pearson correlation analysis was performed to evaluate the correlation among the change of these variables.

**Results:**

After rTMS treatment, the PSQI score was markedly decreased as compared to pre‐rTMS; the concentrations of serum BDNF and GABA were significantly higher; the amplitude of MEPs was markedly reduced. Pearson correlation analysis revealed that the change of PSQI score was negatively associated with the alteration of serum BDNF level and serum GABA level, and positively associated with the change of MEPs amplitude; the change of MEPs amplitude was negatively associated with fold change in the serum BDNF level and the serum GABA level; the increase in serum GABA level was positively associated with the serum BDNF level.

**Conclusions:**

A sequential bilateral low‐frequency rTMS over DLPFC significantly improves primary insomnia probably by increasing the level of BDNF and GABA in the brain and reducing cortical excitability.

## 1 INTRODUCTION

The key characterization of primary insomnia (PI) is dissatisfaction with sleep quantity or quality, which is related to difficulty in falling asleep, maintaining sleep, and/or early morning awakening, affecting the daytime function of patients (Buysse, Rush, & Reynolds, 2017). As the most common sleep disorder, insomnia may seriously threaten quality of life and cause an enormous burden for individuals, families, and the society (Kessler et al., 2012). Due to lack of a clear disease cause, unlike secondary insomnia (e.g., insomnia caused by chronic pain), it is difficult to ameliorate the sleep status of PI patients with treatments. At present, the treatment for PI is still drug‐based. Benzodiazepines have been widely used as a first‐line drug treatment for PI in clinical practice. However, due to patient concerns about side effects of drug treatment and potential dependence, which result in poor medication compliance and therapeutic efficacy, non‐pharmacological treatment has attracted the attention of researchers in the field.

Repetitive transcranial magnetic stimulation (rTMS) is a safe, noninvasive neurophysiological approach. Transcranial magnetic stimulation (TMS) was first applied to evaluate the evoked potential and excitability of the cerebral motor cortex (Barker, Jalinous, & Freeston, 1985). rTMS has been widely used in the treatment of neurological and psychiatric diseases (Shirota, Ohtsu, Hamada, Enomoto, & Ugawa, 2013). In recent years, researchers have found that rTMS has an advantage of improving sleep quality, optimizing sleep structure, and maintaining therapeutic efficacy over pharmacological treatments and cognitive behavioral interventions (Aleman, 2013). rTMS is particularly safe for the treatment of pregnant and lactating women, the elderly, and other special populations with insomnia (Felipe & Ferrão, 2016; Filipčić et al., 2018). However, the optimal rTMS parameters have not yet been determined.

Low‐frequency (1 Hz) rTMS over the right dorsolateral prefrontal cortex (DLPFC) is relatively common at present. It was first used by Kunze, Langguth, Eichhammer, Hajak, and Fleischmann (2007) and found to significantly increase the total sleep duration of insomnia patients. Recently, neuroimaging has also suggested that insomnia patients have higher excitability in the bilateral dorsolateral prefrontal cortex (DLPFC) compared with healthy individuals (Nofzinger, 2004). Moreover, TMS measures showed cortical hyperexcitability in the restless legs syndrome, chronic insomnia, and sleep deprived healthy individuals, but cortical hypoexcitability in the obstructive sleep apnea syndrome (Lanza et al., 2017; Lanza, Cantone, et al., 2018, 2015; Lanza, Lanuzza, et al., 2018, 2015; Lin et al., 2018; Nardone et al., 2013; Salas et al., 2014, 2018). Combined with the inhibitory effect on cortical excitability by low‐frequency stimulation, we speculated that sequential bilateral low‐frequency magnetic stimulation over DLPFC may have a significant therapeutic effect on PI.

At this stage, it is generally believed that rTMS exerts its therapeutic effect on different diseases by modulating cortical excitability and changing neurotransmitter levels in the brain. Increasing evidence indicates that rTMS regulates brain plasticity by promoting the synthesis and release of brain‐derived neurotrophic factor (BDNF; Guo, Lou, Han, Deng, & Huang, 2017). rTMS also promotes the release of gamma‐aminobutyric acid (GABA) in neurons (Baruth et al., 2010; Moisset, Andrade, & Bouhassira, 2016). There is evidence to show that both BDNF and GABA are involved in sleep regulation, and BDNF can promote the function of GABAergic neurons (Mcallister, 2001). However, it is unclear whether sequential bilateral low‐frequency rTMS over DLPFC in PI patients is still effective and how the protocol affects BDNF and GABA levels in the brain.

The inhibitory effect of GABA on nerve cells is mainly achieved by stimulating the GABA_A_ receptor (GABA_A_R), which is a drug target for benzodiazepines against insomnia, anxiety, and withdrawal symptoms (Gielen, Lumb, & Smart, 2012). Animal experiments have shown that increased GABA content in cerebrospinal fluid can prolong the sleep duration of cats (Gottesmann, 2002); whereas rats with rapid eye movement sleep deprivation have increased GABA content in the brain, which is considered an auto‐regulatory mechanism of animals with sleep deficit (Bao, Si, Wang, Wuyun, & Bo, 2016). In sum, both clinical and animal experiments have demonstrated the therapeutic significance of increased GABA content in patients with sleep disorders (Scalise et al., 2014).

BDNF mainly promotes biological effects, such as nerve repair, regeneration, and plasticity formation, through tyrosine kinase receptor B (TrkB; Barco et al., 2005). Kushikata, Fang, and Krueger (1999) showed that injection of exogenous BDNF into the lateral ventricles of rats and rabbits induced sleep, suggesting that increased BDNF levels in the brain may be related to sleep induction. A later study showed that increased BDNF in the brain and the recovery of non‐rapid eye movement sleep in rats with complete sleep deprivation is associated with an increase in cortical slow waves (Faraguna, Vyazovskiy, Nelson, Tononi, & Cirelli, 2008). Maintenance of sleep homeostatic autoregulation in rats with selective sleep deprivation during rapid eye movement sleep is associated with increased levels of BDNF in the body (Datta, Knapp, Koultiwari, & Barnes, 2015), confirming the improvement of insomnia symptoms by BDNF.

In this study, in PI patients treated with sequential bilateral low‐frequency rTMS over DLPFC, the assessment of insomnia symptoms before and after treatment was carried out to observe the therapeutic effects. In addition, changes in the serum GABA and BDNF levels of the patients before and after the treatment were measured to reflect indirectly the GABA and BDNF concentrations in the brain. Finally, further analysis was made on the correlation between the treatment and levels of GABA and BDNF, exploring the therapeutic mechanisms of rTMS for PI treatment.

## 2 MATERIALS AND METHODS

### 2.1 Participants

Thirty‐two eligible right‐handed patients with PI were consecutively recruited as research subjects of this study at the outpatient clinic of Changzhou No. 2 People's Hospital (Xin‐tian, 1983), the Affiliated Hospital of Nanjing Medical University from November 2016 to November 2017. This study was approved by ethics committee of Changzhou No.2 People's Hospital. Inclusion criteria for this study were as follows: (a) met the diagnostic criteria for PI in the third edition of *The International Classification of Sleep Disorders (ICSD‐3)*; (b) PSQI score >7 before treatment; (c) no psychiatric diseases (e.g., depression or anxiety by Hamilton depression or anxiety scale)or neurological diseases (e.g., stroke, dementia, Parkinson's disease, or chronic pain); (d) aged 18–60 years old; (e) an elementary or higher education level; (f) signed the written informed consent form before participating in this study. Exclusion criteria of this study were as follows: (a) had other severe or unstable physical diseases; (b) a history of epileptic seizures; (c) had metal objects (e.g., pacemakers, stents, inner ear hearing aids, or other metal objects) within the body; (d) pregnant or lactating or menstrual or hormone medication women; (e) took psychotropic medications in the past 2 weeks; (f) experienced a major stressful event recently; (g) had severe cognitive impairment, language barrier, or inability to cooperate with the treatment; (h) structural brain lesions by cranial MRI scan (except most patients with a recent cranial MRI scan, the remaining patients were requested to perform the scan). None of the participants were on opioids, sedative hypnotics, antipsychotics, antidepressants, β‐blockers, stimulants, mood stabilizers, or medications for thyroid/diabetes. Patients were not permitted to use alcohol, caffeine, or tobacco at any time during this study. The recruitment of patients was performed by an experienced neurologist.

### 2.2 TMS protocol

Repetitive transcranial magnetic stimulation was delivered by a commercially available magnetic stimulator (CCY‐IV model; YIRUIDE Inc, Wuhan, Hubei province, China) with a 70‐mm figure‐of‐eight coil and an electromyography device, and with a frequency of 0–100 Hz, maximum output magnetic field strength of 1.5–6 T, and 80%–120% MT stimulation intensity. Each participant was allowed to sit in a reclining chair with their hip and knee joints at 90°. According to a magnetic stimulation cap designed on the basis of the International 10–20 electroencephalography system, resting motor threshold (rMT) of the left or right abductor pollicis brevis (APB) muscle was measured to determine the motor evoked potentials (MEPs) of the left or right primary motor cortex following surface electrodes were placed on the left or right APB muscle. rMT was measured as the minimal stimulus intensity required to produce MEPs of at least 50 µV in at least 5 of 10 consecutive trials. Stimulation intensity for the rTMS procedure was set at 80% of the rMT. The amplitude of the MEPs evoked by rTMS was measured peak‐to‐peak (Nojima & Iramina, 2018). The stimulation site of the left or right DLPFC was 5‐cm anterior to a “hot spot” for producing the maximal motor response in the left or right APB muscle. The coil was held tangential to the scalp with the handle pointing at the occipital side of the scalp. The specific treatment parameters of this study as described in our previous work (Lu et al., 2018) were 1 Hz stimulation frequency, 10 s stimulation, 2 s inter‐interval, stimulation intensity was set at 80% of rMT (a subthreshold stimulation theoretically leading to an inhibitory effect), 30 min stimulation duration (first 15 min stimulation over left DLPFC, and then 15 min stimulation over right DLPFC), a total of 1,500 pulses, one session per day, 5 days per week for 2‐week treatment course (with 2 days off each weekend). All participants received the same treatment parameters. The delivery of rTMS procedure was scheduled in the morning.

### 2.3 Clinical assessments

Each subject received PSQI evaluation before treatment and after treatment. The PSQI has been widely recognized by researchers around the world (Riemann et al., 2010). The PSQI is used to assess mainly self‐reported sleep quality in sever aspects: subjective sleep quality, sleep onset latency, sleep duration, habitual sleep efficiency, sleep disorder, consumption of sleeping medication, and daytime function. PSQI scores range from 0–21, with a self‐reported score >7 considered sleep disorder; the higher the PSQI score, the worse the sleep status. In addition, Epworth sleepiness scale before and after rTMS，and excluded patients with excessive daytime sleepiness into this study.

### 2.4 Measures of serum level of BDNF and GABA

All participants were recruited on Monday morning at the outpatient department. After clinical assessment, 3 ml of peripheral venous blood was collected from each eligible participant. They received rTMS treatment in the morning. After the whole procedure of rTMS, we made clinical assessments. About 1 hr later, 3 ml of blood sample of patients was collected again. Three milliliters of peripheral venous blood were collected from each subject before treatment and at the end of the treatment and were placed in EDTA anticoagulation tubes to allow standing at room temperature for 1 hr, followed by centrifuging at 3,000 *g* at 4°C for 8 min. One milliliter of serum in the supernatant was collected and preserved at −80°C before testing. The serum BDNF level of each subject before and after the treatment was determined by an ELISA kit strictly in accordance with the manufacturer's instructions (Hangzhou LianKe biotechnology Co. Ltd., Hangzhou, Zhejiang Province, China).

The venous blood samples were let stand and centrifuged as described in the previous subsection to collect 1 ml serum and preserved at −80°C for later experiments. A Waters ACQUITY ultra‐high performance liquid chromatography (UPLC) system was used for GABA chromatography. The columns used were Merck SeQuant Zic‐HILIC hydrophilic columns. The isolated GABA was subjected to mass spectrometry analysis using an AB SCIEX Triple Quad 5500 mass spectrometer (Applied Biosystems, Shanghai, China).

### 2.5 Statistical analysis

GraphPad Prism 6.0 software (GraphPad Software, La Jolla, CA) were used for statistical analysis in this study. The measurement data were presented as mean ± standard deviation (*SD*). Differences in PSQI scores as well as serum BDNF and GABA levels before and after the treatment were analyzed by pairwise *t* test. Pearson's correlation analysis was used to evaluate the correlation between the changes in PSQI and serum BDNF and GABA levels after the treatment. *p* < 0.05 was considered a statistically significant difference.

## 3 RESULTS

### 3.1 General information of research subjects

According to the above inclusion criteria, 32 subjects (12 men and 20 women) without anxiety, depression, stroke, or other neurological or mental illness, who had not consumed psychotropic drugs recently and had pretreatment PSQI scores of 15.84 ± 3.72, were included in this study. The clinical baseline data and statistics of the patients, including sex, age, education level, marital status, work status, disease duration, and comorbidity, are shown in Table 1.

**Table 1:** Participants characteristics

	Participants (*n* = 32)	*n*%
Sex		
Male	12	37.5
Female	20	62.5
Age (years), median (IQR)	44.8 (25–62)	
Education		
Primary	6	18.6
Secondary	10	31.2
University	16	50
Marital		
Married	28	87.5
Single	4	12.5
Work status		
Employed	21	65.6
Unemployed	3	9.4
Retired	8	25
Duration of illness (years), median (IQR)	3.5 (1–8)	
Number of somatic illnesses		
None	17	53.1
One	9	28.1
Two or more	6	18.8

Note

Nine patients had stable somatic diseases, such as hypertension or hyperlipidemia, or diabetes. Six patients suffered from the combination of these diseases. Before the recruitment, these patients had a stable control of blood pressure, and/or blood lipid, and/or blood glucose by regular medications. During treatment, the blood variables had no significant fluctuation.

### 3.2 Changes in PSQI score, the serum level of BDNF and GABA, and MEPs after rTMS treatment

**Figure 1:**
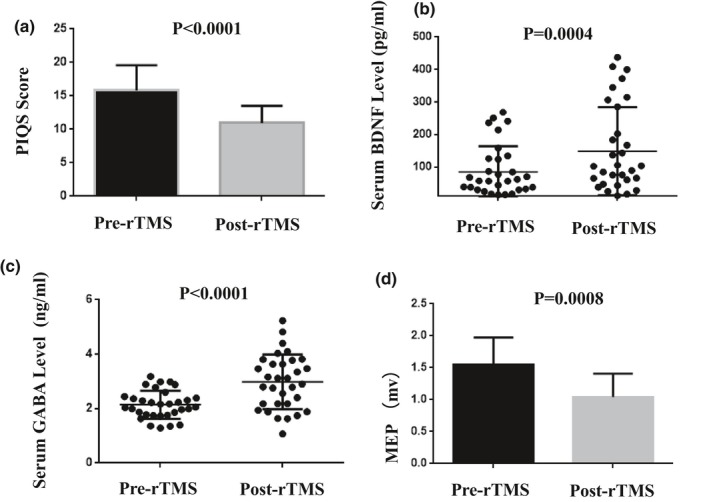
The PIQS score, serum BDNF concentrations, serum GABA concentrations, and MEPs before and after rTMS. *p* Value is shown in the Figures, as compared to pre‐TMS. BDNF: brain‐derived neurotrophic factor; GABA: gamma‐aminobutyric acid; MEPs: motor evoked potentials; rTMS: repetitive transcranial magnetic stimulation

The pre‐ and post‐treatment PSQI scores of the patients were 15.84 ± 3.72 and 10.94 ± 2.58, respectively, suggesting that the PSQI scores of the patients were significantly reduced 10 days after the rTMS (*p* < 0.0001, Figure 1a). Moreover, there was no significant change in the score by Epworth sleepiness scale between before and after rTMS. The serum level of BDNF after rTMS treatment was significantly elevated (148.67 ± 134.97 pg/ml vs. 85.20 ± 78.77 pg/ml, *p* = 0.004, compared to pre‐rTMS, Figure 1b). Similarly, the serum GABA level of the patients after rTMS treatment was remarkably increased (2.98 ± 1.00 ng/ml vs. 2.15 ± 0.51 ng/ml, compared to pre‐rTMS, *p* < 0.0001, Figure 1c). In contrast, the amplitude of MEPs reflecting the left cortical excitability was markedly reduced (1.04 ± 0.36 mV vs. 1.55. ± 0.42 mV, compared to pre‐rTMS, *p* = 0.0008, Figure 1d).

### 3.3 Correlation between PSQI Improvement and the change in the serum BDNF and GABA Level and MEP alterations after rTMS treatment

**Figure 2:**
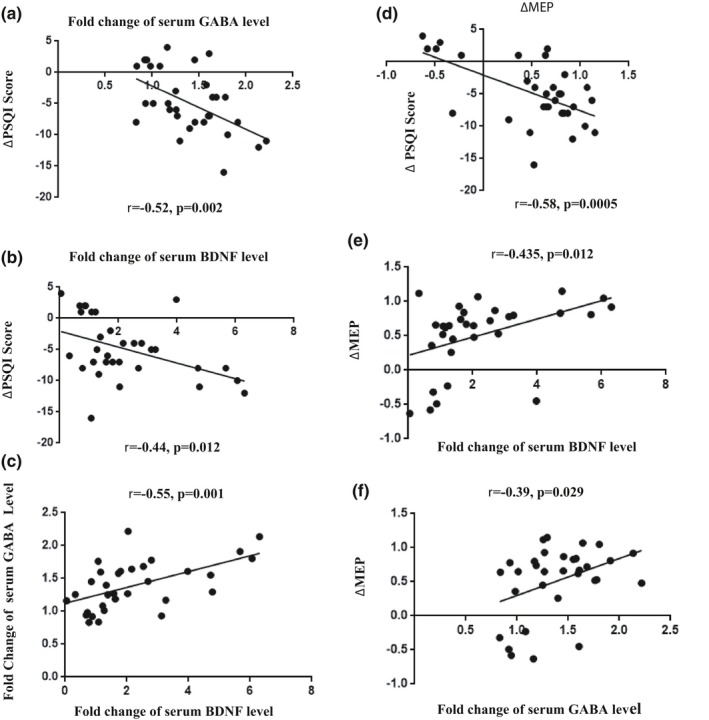
The correlations of the change of the PIQS score, serum BDNF, GABA concentration and MEPs. The value of *r* and *p* is shown in the figures. BDNF: brain‐derived neurotrophic factor; GABA: gamma‐aminobutyric acid; MEPs: motor evoked potentials

Pearson's correlation analysis on the relationship between fold change in the serum GABA level and the difference in PSQI scores (post‐treatment PSQI–pre‐treatment PSQI, ΔPSQI hereafter) before and after the treatment showed a negative correlation (*r *= −0.52, *p = *0.002, Figure 2a).

Similarly，a negative correlation was observed between ΔPSQI and fold change in the serum BDNF level (*r *= −0.44, *p = *0.012, Figure 2b). But there was a positive association between fold change of the serum GABA level and fold change of the serum BDNF level (*r = *0.55, *p = *0.001, Figure 2c). Next, Pearson's correlation analysis revealed that ΔMEP was positively related to ΔPSQI (*r = *0.58, *p = *0.0005, Figure 2d) and negatively associated with fold change in the serum BDNF level (*r = *0.435, *p = *0.012, Figure 2e) and the serum GABA level (*r = *0.35, *p = *0.029, Figure 2f).

## 4 DISCUSSION

Although pharmacological treatment has been the main approach for PI, rTMS has received much attention due to its noninvasiveness, painlessness, effectiveness, ease of operation, and safety. In this study, we found that sequential bilateral low‐frequency rTMS over DLPFC alleviated primary insomnia, reduced the amplitude of MEP, and increased the serum level of BDNF and GABA. Further, we showed their correlations. These finding suggested that this procedure significantly ameliorates primary insomnia probably by increasing the level of BDNF and GABA in the brain and reducing cortical excitability.

To date, there have been some studies on rTMS for the PI treatment, but relatively specific and well‐recognized therapeutic regimens are not available. In major depressed patients, hypoactivity in the left DLPFC and relative hyperactivity of the right DLPFC were observed by functional MRI (Grimm et al., 2008). Thus, low‐frequency (1 Hz) stimulation over right DLPFC or high‐frequency stimulation over left DLPFC or sequential bilateral rTMS was used for depression treatment or drug craving (Chen et al., 2013; Fitzgerald et al., 2006; Liu et al., 2017). In this study, we conducted a sequential bilateral low‐frequency stimulation of DLPFC in patients with PI as a therapeutic regimen based on the increase of cortical excitability of bilateral DLPFC detected by neuroimaging and TMS measure (Lanza, Cantone, et al., 2015; Lin et al., 2018; Nardone et al., 2013; Salas et al., 2018). By comparing the PSQI changes before and after the treatment, we found that bilateral low‐frequency rTMS significantly reduced the PSQI score of PI patients, suggesting a good therapeutic effect on PI. In addition, serum BDNF and GABA levels of the PI patients were significantly elevated after the treatment. The differences in serum BDNF and GABA levels were significantly correlated with the changes in PSQI after the treatment, suggesting that BDNF and GABA levels in the brain may be associated with the therapeutic mechanism of sequential bilateral low‐frequency rTMS in PI.

On one hand, studies on the effects of rTMS on depression and autism have shown that specific sites and frequencies of rTMS promote the release of endogenous GABA as well as reduce the loss of GABA_A_R on the postsynaptic membrane, thereby increasing the efficacy of GABAergic neurons (Dubin et al., 2016; Tan et al., 2018). Similarly, studies of rTMS in cognitive impairment and dyskinesia after stroke have shown that rTMS has an effect on the BDNF‐TrkB receptor pathway in the brain (Guo et al., 2017; Luo et al., 2017). On the other hand, neuroimaging and TMS measure showed cortical hyperactivity in the several types of sleep orders (Lanza, Cantone, et al., 2015; Lin et al., 2018; Nardone et al., 2013; Salas et al., 2018). Thus, the inhibitory rTMS would dampen the excitability. Our results confirmed that low‐frequency subthreshold rTMS decreased MEP amplitude reflecting the motor cortical excitability (Magalhães et al., 2015) in the PI patients. Consistently, Lanza G et al. reported that low‐frequency (1 Hz) rTMS reduced MEPs amplitudes in healthy control and patients with RLS, with a more pronounced significance in controls, suggesting impaired cortical plasticity in RLS patients. (Lanza, Lanuzza, et al., 2018). Cortical hyperactivity is often correlated to the enhancement of excitatory neurotransmitter system (such as glutamate transmission). Recently, there is evidence that low‐frequency rTMS over auditory cortex of the left temporal lobe for the treatment of tinnitus resulted in a down regulation in the excitatory glutamate neurotransmitter of bilateral auditory cortical areas measured by single voxel proton magnetic resonance spectroscopy, which highly associated with a reduction of loudness levels (Cacace et al., 2017). Conversely, using the same transmitter‐measured methods, Dlabac‐de Lange et al. (2017) reported that bilateral high‐frequency rTMS over prefrontal cortex increased glutamate and glutamine concentrations in left prefrontal cortex of schizophrenia patients with predominant negative symptoms. Together, frequency‐dependent rTMS may bi‐directly modulate the excitatory glutamate system and affect cortical excitability, eventually exerting its therapeutic effects.

Regarding the therapeutic mechanism of rTMS for PI, several explanations are currently available. First, a large number of basic and clinical studies have indicated that the cerebral cortex of PI patients is in an excessively awakened state, which leads to excessive hyperactivities of the hypothalamic–pituitary–adrenal axis and hypothalamic–pituitary–thyroid axis function in the body, resulting in elevation of serum corticosterone, adrenocorticotrophic hormone, thyrotropin, and free T3 and T4 levels. Therefore, serum concentrations of these hormones indirectly reflect the cortical arousal level to some extent (Riemann et al., 2010; Vargas et al., 2018). Based on this, some researchers have shown that low‐frequency rTMS on the right DLPFC reduces the levels of adrenocorticotrophic hormone, thyroid‐stimulating hormone (TSH), and free T3 and T4 levels in the serum of PI patients (Jiang, Zhang, Yue, Yi, & Gao, 2013), suggesting that low‐frequency TMS may reduce the excitability of the cortex. Combined with the therapeutic principle of rTMS, we speculated that low‐frequency magnetic fields induced hyperpolarization of cortical neurons and reduced the metabolism and excitability of the corresponding cortex, thereby playing a role in improving sleep. Thus, we selected sequential bilateral low‐frequency magnetic stimulation as a therapeutic regimen in this study to achieve a significant effect, possibly due to a wider range of cortical inhibition. Second, rTMS exerts a protective effect in the brain by reducing neuronal oxidative stress, especially lowering the damage to neurons in the hippocampus (Guo et al., 2017) which is believed to be an important brain structure related to sleep. In addition, rTMS has been shown to promote melatonin secretion in the pineal gland (Strafella, Paus, & Dagher, 2003), thereby regulating the sleep/arousal cycle. rTMS also regulates the levels of various neurotransmitters and neuromodulators in the brain, such as serotonin and norepinephrine, maintains the balance between excitatory neurotransmitters, and inhibitory transmitters, thus improving the symptoms of insomnia (Nakamura & Nagamine, 2017; Sivolap, 2017). In this study, we measured the serum BDNF and GABA levels to indirectly reflect the GABA and BDNF levels in the brain. Our results showed that rTMS increased serum BDNF and GABA concentrations in PI patients. The changes in serum BDNF and GABA concentrations after the treatment were associated with the improvement of insomnia symptoms and the decreased cortical excitability as reflected by MEPs. Therefore, we speculated that rTMS may exert its effect by increasing the level of BDNF and GABA in the brain and further regulate cortical excitability. Moreover, there is evidence to show that BDNF can promote the function of GABAergic neurons (Mcallister, 2001). Our data revealed a positive association between the change of serum BDNF and serum GABA. These suggested that increased serum GABA level may be at least partly attributed to enhanced serum BDNF level.

Limitations of this study included (a) we should make conclusions with carefulness due to an open‐label trial with a small sample size; (b) a control group and a sham stimulation group were not included in this study, so a strict control trial will be required to exclude placebo effects; (c) we did not perform follow‐up evaluation after the treatment and therefore could not provide evidence on the sustained rTMS therapeutic effects on PI; (d) the wide age range may influence the homogeneity of the sample; (e) we failed to perform polysomnographic monitoring, and as a result could not completely exclude other sleep disorders; (f) there is no direct evidence showing the BDNF and GABA levels in the brain. The BDNF and GABA levels determined in this study were based on the levels in the peripheral venous blood, which might potentially affected by other factors and thus were only an indirect reflection of BDNF and GABA levels in the brain.

## CONFLICT OF INTEREST

The authors declared no conflict of interest.

## AUTHORS’ CONTRIBUTIONS

XZ conceived and developed this study. JF, QZ, CZ performed the experiments. XZ and ZW discussed and analyzed the data and wrote the manuscript. All authors read and approved the final manuscript.
